# Design and Testing of Real-Time Sensing System Used in Predicting the Leakage of Subsea Pipeline

**DOI:** 10.3390/s22186846

**Published:** 2022-09-09

**Authors:** Yung-Hsu Chen, Sheng-Chih Shen, Yan-Kuei Wu, Chun-Yen Lee, Yen-Ju Chen

**Affiliations:** Department of Systems and Naval Mechatronic Engineering, National Cheng Kung University, Tainan 70101, Taiwan

**Keywords:** hall sensors, leakage sensing system, subsea pipeline, deep-sea water

## Abstract

This study integrates the array sensing module and the flow leakage algorithm. In this study, a real-time monitoring deep-sea pipeline damage sensing system is designed to provide decision-making parameters such as damage coordinates and damage area. The array sensor module is composed of multiple YF-S201 hall sensors and controllers. YF-S201 hall sensors are arranged inside the pipeline in an array. The flow signal in the deep-sea pipeline can be transmitted to the electronic control interface to analyze the leakage position and leakage flowrate of the pipeline. The theory of this system is based on the conservation of mass. Through the flow of each sensor, it is judged whether the pipeline is damaged. When the pipeline is not damaged, the flowrate of each sensor is almost the same. When the pipeline is damaged, the flowrate will drop significantly. When the actual size of leakage in the pipeline is 5.28 cm^2^, the size calculated by the flowrate of hall sensors is 2.58 cm^2^ in average, indicating the error between experimental data and theoretical data is 46%. When the actual size of leakage in the pipeline is 1.98 cm^2^, the size calculated by the flowrate of hall sensors is 1.31 cm^2^ in average, indicating the error between experimental data and theoretical data is 21%. This can accurately confirm the location of the broken pipeline, which is between sensor A and sensor B, so that the AUV/ROV can accurately locate and perform pipeline maintenance in real time. It is expected to be able to monitor the flowrate through the array magnetic sensing module designed in this study. It can grasp the status of deep-sea pipelines, improve the quality of deep-sea extraction and pipeline maintenance speed.

## 1. Introduction

The study used hall sensors to form a monitoring system to detect leakage of deep-sea pipelines on the seafloor. Deep-sea pipelines play an important role in human life because of the advantages of low cost and no environmental restrictions. Therefore, how to design a high-accuracy damage detection system is worthy of discussion. There are some techniques for detecting leakage, such as ultrasound and optical fibers. Muhammad Muzakkir Mohd Nadzri [1] uses long range ultrasonic testing (LRUT) to detect the condition of a pipeline. LRUT has the capability of structural safety monitoring and is often used in pipeline inspection. As a non-destructive testing (NDT), LRUT has the ability to quickly screen and detect various types of defects. Joseph D Butterfield [2] discovered leakages through leak noise correlation by placing sensors on either side of the leak and recording and analyzing its vibroacoustic emissions. The purpose of the study was to predict leakage flowrate in pipelines using vibroacoustic emission signals. The study found that both the leakage velocity and the leakage area affect the leak spectrum. Different features were obtained from the raw signal, which were analyzed to accurately predict the leakage flowrates without prior knowledge of the pore area. The leakage area can also be accurately predicted without prior knowledge of the leakage flowrate. The Ahmed Atef [3] method of detecting and locating leakages in water distribution networks using ground penetrating radar (GPR) and infrared photography (IR). The method was successfully applied to detect simulated leakages and real leakages. The error is small (2.9–5.6%) in estimating the leakage area, so it can help operators to detect and locate water leakages with high accuracy. A fiber optic sensor has the advantage of being immune to electromagnetic interference, and has multi-point sensing points, but the structure of fiber optic sensing is not strong. Fiber optic sensors need to design a special encapsulation protection structure. Davis [4], in 2013, studied measurement techniques for a subsea pipeline ring strain in Gulf of Mexico oil fields. K.S. Ong [5] developed a simple-to-fabricate and low-cost acoustic vibration sensor based on optical fiber (SMF-28). Optical fiber sensors consist of a bending structure and use bending loss as a sensing mechanism to detect leakages in pipelines. A measurement system for the optical fiber sensor is proposed. Jia Zhang [6] applies distributed temperature sensing (DTS) and localization methods using temperature signals to pipeline networks. However, it is difficult for DTS to comprehensively monitor pipeline leakage, and DTS has monitoring blind spots. Continuous low temperature changes are difficult to capture by optical fibers, so distributed acoustic sensing (DAS) is required to assist in comprehensive monitoring. The amplitude attenuation model of leakage sound is established, and the law of vibration sound signal in porous soil is studied. When the pipeline leaks, the amplitude of the time domain signal and the vibration is obvious. Sonic leakage occurs in a very short period and the vibration amplitude peaks. Ma Yi-lai [7] analyzes the magnetic circuit of the magnetization structure of the intelligent magnetic flux leakage detection robot, using the mechanical structure to adjust the motion posture to meet the detection requirements.

In this paper, several hall sensors were used to build up a monitoring system to detect the leakage in subsea pipeline. Advantages such as low-cost and being free from environmental restrictions make subsea pipeline an important role in human life [8,9]. This method uses the characteristics of the flow field to determine whether there is damage. It is currently widely used in flowrate detection and water flowrate monitoring systems. Therefore, this study hopes to use the hydraulic leak detection method to accurately detect the damaged area and location of deep-sea pipelines; however, factors such as earthquakes and corrosion can cause broken pipelines and lead to damage to the marine ecosystem and economy [10,11,12,13]. The American scientific institution pointed out that the efficiency of most leakage detection systems is only 20% [14]. Therefore, designing a system which can detect the leak accurately is a challenge waiting to be overcome. Today, several techniques applied to leakage detection include ultrasonic-guided wave and optical fiber. The common disadvantages of these techniques are high manufacturing cost and the fact that accuracy is influenced by the shape and material of the pipelines [15]. To address these disadvantages, the hydraulic leak detection method, which uses flow distribution to detect a potential leak in the pipeline, was developed in 1960 [16]. Among most hydraulic leak detection methods, some researchers used hydraulic pressure to detect the leak. However, this method is more suitable for finding larger leaks in the pipeline [17]. Therefore, the aim of this research is to use YF-S201 hall sensors to detect the flowrate and find the leak in the pipeline. The YF-S201 hall sensor consists of a hall IC on the gear. When the water flows through the sensor, the gear will rotate and lead to the variation of magnetic field, making the hall effect happen; then, the IC will signal output, and the computer uses this signal to calculate the flowrate. The hall sensor is widely used in flowrate detecting [18] and water flow monitoring system [19] because of its low cost. In addition, the sensor equipped on the gear has high accuracy, and statistics show that this kind of sensor is one of the most accurate flow sensors. The hall sensor detects the flowrate of each water intake pipeline. The hall sensor is a turbine-based sensor and can roughly determine the damaged position and area of the pipeline by the flowrate change. Sood [18] measures the water flowrate in the irrigation pipeline through the hall sensor, and determines whether there is a water leak in the irrigation pipeline by detecting the water flowrate. In order to reduce the waste of water resources and achieve high-efficiency application, Kolhare [20] developed a microcontroller-based turbine flowmeter system to measure water flowrate in solar water heaters. The rotational pulse is generated by the turbine rotor, magnet, and hall effect sensor, and the flowrate can be converted by calculating the number of pulses per minute. Sinha [21] has developed a set of non-contact flowrate measurement techniques using hall effect sensors and rotameters. They measured the change in the magnetic field through a hall-effect sensor placed outside the rotameter and converted the change into a DC signal. Finally, the DC signal is transmitted to the computer, and the water flowrate is calculated through theoretical equation.

In recent years, many hi-tech flowrate sensors have been developed; when using the sensor, many researches combine sensors with machine learning, which tries to predict the accuracy of data. In terms of leakage detection systems, some researchers use spherical detectors to measure the leak sound from the inside of pipeline to detect the leakage, the system also combined with the SVM model, which makes the accuracy of the system up to 93% [22]. Some researches detect lots of parameters, such as pressure, temperature, and flowrate, then combine all the data with machine learning to determine the leakage [23]. There is also the raman distributed fiber sensor, which uses the dynamic threshold identification method to measure the distributed temperature to detect the pipeline leak, and results shows that the positioning accuracy is 1 m [24]. However, these methods are too expensive to be widely used in long subsea pipeline. The system is also complex and the machine learning may take lots of time to design and train. The machine learning model will also be different when used in different pipeline. Therefore, this research aims to use a low-cost magnetic module based on YF-S201 hall sensors to accurately detect leakage in subsea pipeline; a system detects the change of flowrate in the pipeline, and is one of the hydraulic leak detection methods. The system consists of several YF-S201 hall sensors, and an Arduino Uno and computer, which is timesaving and easily implemented in most subsea pipeline. In this paper, the result will show the data of each YF-S201 hall sensor and prove the feasibility of this method by utilizing that data. A crack estimation algorithm is established in the research, and practical verification experiments are designed. In the future, the system can be widely used in pipelines to improve deep-sea water quality.

## 2. Materials and Methods

**Figure 1:**
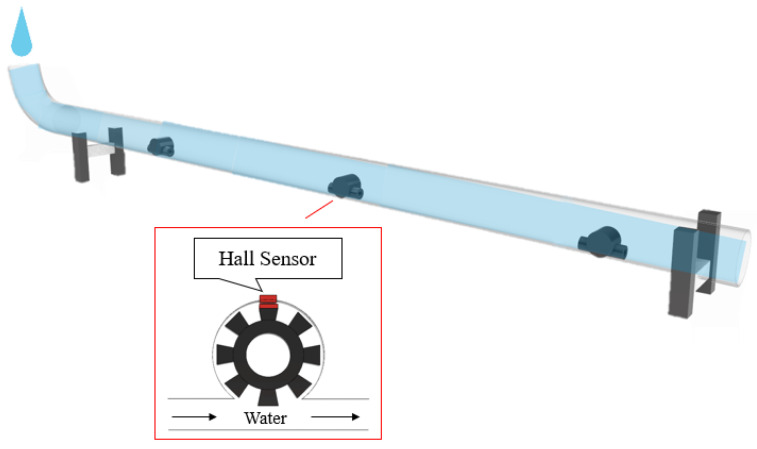
Schematic of hall sensors in the pipeline filled with water.

**Figure 2:**
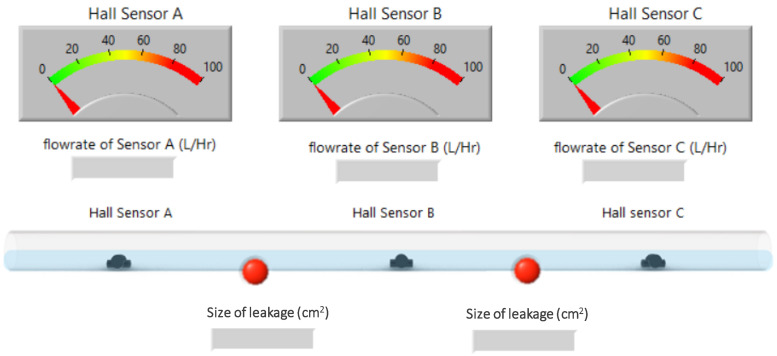
Human-machine interface of subsea pipeline detection system.

The research used an acrylic tube filled with water to simulate the subsea pipeline, as Figure 1 shows. There are three YF-S201 hall sensors in the acrylic tube to detect the flowrate in the pipeline. When water flows through the hall sensor, the hall IC on gear rotates and outputs PWM signal. The software can use the PWM signal to calculate the flowrate. The research designed a LabVIEW human-machine interface, shown in Figure 2, enabling researchers to see the situation of the subsea pipelines. The interface is a real-time monitoring interface, which shows the flowrate of each sensor per second. The interface is easy to use; when the sensors connect to Arduino Uno, the software will calculate the flowrate and show the location and the size of leakage on the LabView interface. In addition, the red warning light in the interface will light up when there is any leakage. By doing so, the researcher can easily keep track of the situation of the subsea pipelines. Arduino Uno is applied to read the PWM signals from hall sensors in this study. Besides, a LabVIEW human-machine interface is also designed to monitor the real time situation from the hall sensors in the pipeline. The hall sensor can determine the frequency of the flowrate in the pipeline in a period of time and output PWM signals. The output PWM signals will be received by Arduino Uno and converted to voltage. The ultimate voltage will then be shown on the LabVIEW human-machine interface, where we can monitor the condition of each sensor in the pipeline in real time.

**Figure 3:**
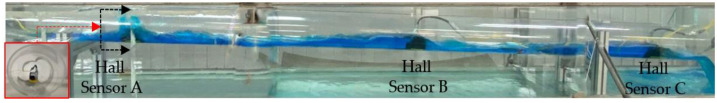
Experimental equipment of miniaturized subsea pipeline with hall sensors.

Figure 3 shows the experiment setup. In the experiment, there is a 250 cm long acrylic tube filled with water and three YF-S201 hall sensors which are placed in a row. The sensors from the left to right are Sensor A, Sensor B, and Sensor C. The diameter of the tube is 10 cm, and the height of the hall sensor is 3.5 cm. The w90er is poured into the tube from the left side of Sensor A with flowrate about 920 L/h, 1300 L/h, and 1951 L/h, and the pipeline is inclined slightly to avoid the reflow of water. The research also added some blue ink into the water to make the flow field obvious in experiments. Furthermore, in order to stimulate leakages in the pipeline, the research used an electric drill to drill some holes in the tube between Sensor A and Sensor B; the diameter of the holes are 9.4 mm, 9.7 mm, 8.4 mm, and 20.5 mm, respectively. When doing the experiment, we used waterproof tape to adjust the size of hole.

The accuracy of the hall sensor is only 90%, therefore, the size of hole needs to be big enough to be detected. In the following experiments, we decided the size of the holes should be 1.98 cm^2^ and 5.28 cm^2^. With the experiment set up, this research aims to verify the feasibility of the system for using hall sensors to detect leaks in subsea pipelines.

The voltage-related, frequency-related, and flowrate-related, are given by the following relations:

(1) $$\text{The voltage of PWM signal}=\left(\text{making time}/\text{total time}\right)\;\times \;\text{max}\;\text{voltage}$$

(2) $$\text{Flowrate of hall sensor}\left(\text{L}/\text{hour}\right)=\;\frac{\left(\text{Gear rotational frequency}\times 60\right)}{7.5}$$

Therefore, this research uses the PWM signal to calculate flowrate in the pipeline.

The research takes the pipeline as a system and lets the control volume surround the pipeline. The research uses the law of conservation of mass to detect the leakage; the equation is as follows:

(3) $$\frac{D}{Dt}\int_{sys}\rho \;\mathrm{dV}=\rho \;\mathrm{dV}+\int_{cs}\rho V\;\hat{n}dA=0$$

where A is the cross-sectional area of pipeline, ρ is the density of the fluid, *V* is control volume, V is average velocity of the fluid, *Q* is flowrate, and $\dot{m}$ is mass flowrate.

**Figure 4:**
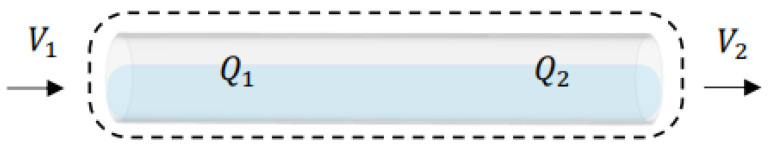
Schematic of pipeline and parameters.

Assume the flow field in the pipeline is stable. Based on Equation (3), when there is no leakage in the pipeline, as Figure 4 shows, the system can use Equation (4) as follows:

(4) $$Q_{1}=Q_{2}=Q_{3}$$

where Q stands for flowrate in the pipeline.

**Figure 5:**
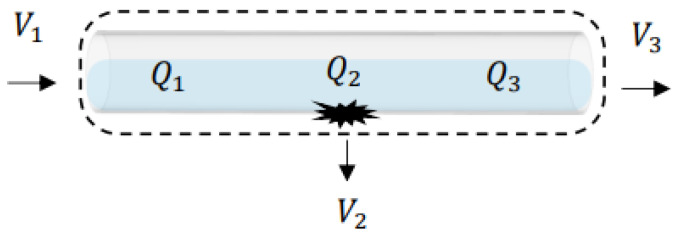
Schematic of broken pipeline and parameters.

Assume the flow field in the pipeline is stable as before. When there is a leak in the pipeline, as Figure 5 shows, the system can use Equation (5) as follows. Using Equation (5), the leak can be found from the flowrate.

(5) $$Q_{1}=Q_{2}+Q_{3}$$

**Figure 6:**
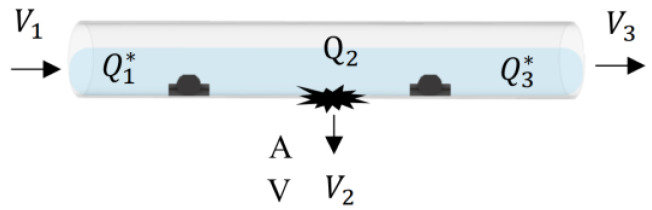
Schematic of broken pipeline with hall sensors and parameters.

Then, we put several hall sensors in the pipeline, as Figure 6 shows. According to many experiments, the research found that hall sensors only detect 4.7% flowrate in the pipeline, as Table 1 shows.

According to the result of Table 1, the research uses Equation (6) to calculate the flowrate of wate $Q_{2}$ through the leakage:

(6) $$Q_{2}=Q_{3}^{*}-Q_{1}^{*}/0.047$$

where Q^*^ stands for the flowrate of hall sensors.

**Figure 7:**
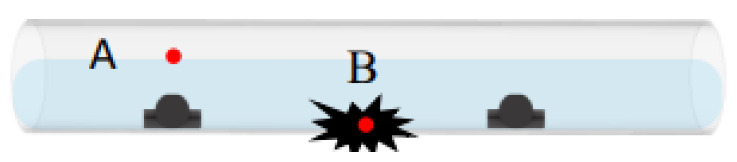
Schematic of broken pipeline with hall sensors.

After we found out the location of the leak, in order to find the size of leakage, the research uses Bernoulli’s principle to find the water velocity $V_{B}$ at the leak site, as shown in Figure 7. The Bernoulli’s principle is shown as Equation (7).

(7) $$\frac{P_{A}}{r}+Z_{A}+\frac{V_{A}^{2}}{2g}=\frac{P_{B}}{r}+Z_{B}+\frac{V_{B}^{2}}{2g}$$

where *P* stands for pressure, r for specific weight of water, *Z* for the water level, and *V* for the velocity of water.

The fact that point *A* and *B* contact air allows us to view the pressure of point *A* and *B* as zero, as shown in Equation (8).

(8) $$P_{A}=P_{B}=0$$

According to the location of point *B*, the height of point *B* was considered as zero, as shown in Equation (9).

(9) $$Z_{B}=0$$

Therefore, in this research, $V_{B}$ was calculated by the parameter above and then used to calculate the size of leakage by $V_{B}$

(10) $$A_{leakage}=Q_{2}/V_{B}$$

## 3. Results

**Figure 8:**
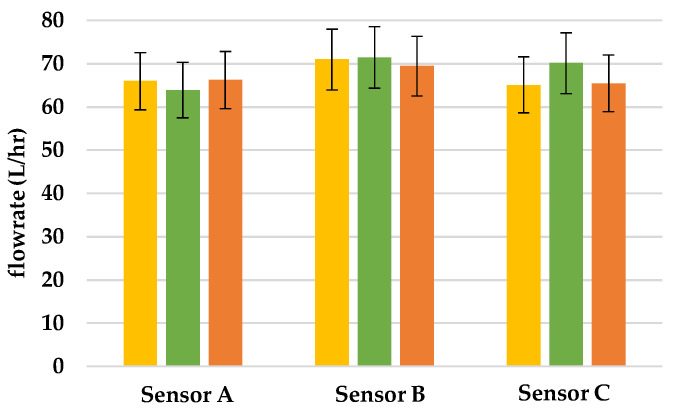
Flowrate of sensors when there is no leakage in the pipeline.

**Figure 9:**
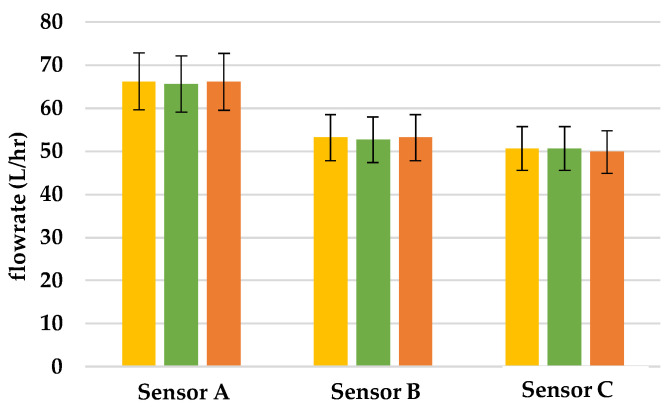
Flowrate of sensors when there is 1.98 cm^2^ leakage in the pipeline.

**Figure 10:**
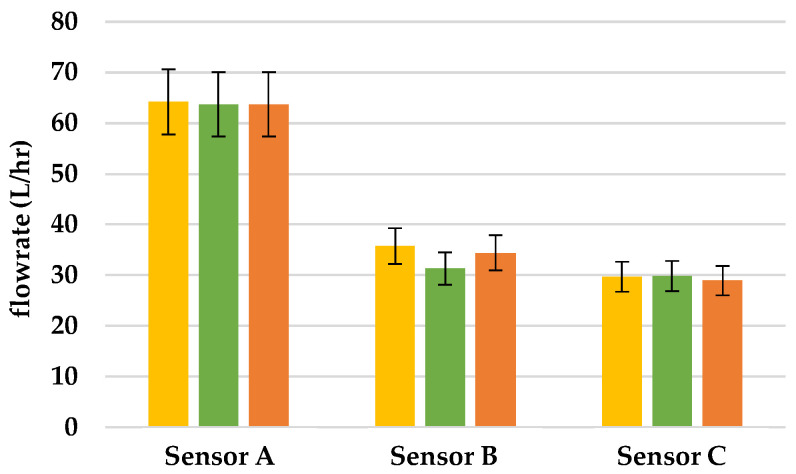
Flowrate of sensors when there is 5.28 cm^2^ leakage in the pipeline.

**Figure 11:**
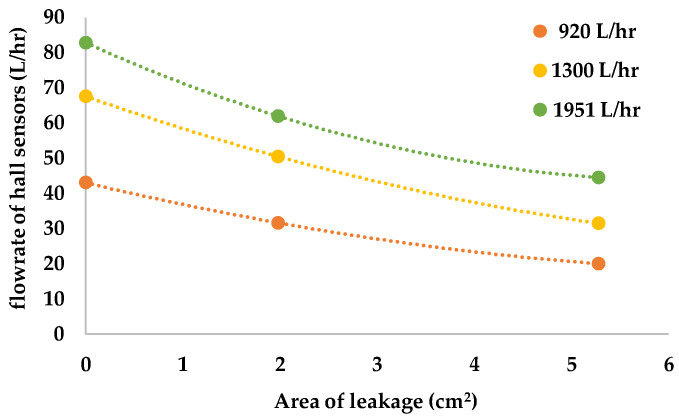
Variation of flowrate of hall sensors with respect to different size of leakage, for different flowrate in the pipeline.

This research was intended to verify if the flowrate of hall sensors changes when there is leakage in the pipeline. We put Sensors A, B, and C, in the pipeline and poured water into the tube from the left of Sensor A. In this research, each experiment was conducted three times and in Figure 8, Figure 9 and Figure 10, we used yellow, green, and orange bar charts, to show the result of each experiment. In addition, we also used all data to draw Figure 11, to show the relationship between each result.

### 3.1. Finding the Location of Leakage

The total flowrate in the pipeline is 1300 L/h and Figure 8, Figure 9 and Figure 10 show the flowrate that the hall sensors detected under different sizes of leakage in the pipeline. In Figure 8, the average flowrate of three hall sensors is 66 L/h in average. According to the datasheet, the accuracy error of the YF-S201 hall sensor is 10%; therefore, the flowrate of the three hall sensors shown in Figure 8 can be considered as the same. The result of the experiment shows that the flowrate of the hall sensors is approximately the same when there is no leakage in the pipeline.

To simulate leakage in the pipeline, we made several holes approximately 100 cm away from the mouth of the pipeline and poured 1300 L/h flowrate of water into pipeline. According to Figure 9, when there is 1.98 cm^2^ leakage between Sensors A and B, the flowrate between Sensor A was the same as in Figure 8; however, the flowrate between Sensors B and C declined by 23%, due to the leakage in the pipeline. Thus, through this system, we can determine that there is a leakage between Sensors A and B, and no leakage between Sensors B and C. When there is 5.28 cm^2^ leakage between Sensors A and B, the flowrate of Sensor A was the same as Figure 8; however, the flowrate of Sensors B and C declined by 53% due to the leakage in the pipeline, as shown in Figure 10. Based on this finding, we can tell that there is a greater leakage between Sensors A and B, and no leakage between Sensors B and C. 

Besides from the experiments in Figure 8, Figure 9 and Figure 10, we also did experiments when the flowrate of the pipeline was 920 L/h and 1951 L/h, and Figure 11 shows all the relationships of those data. Through Figure 11, we can see the relationship between hall sensor flowrate and the size of the leakage when the flowrate in the pipeline is different. The result showed that the bigger the leakage is, the more it declines. Therefore, the experiment proved that the system can use the flowrate of hall sensors to determine the variation of the size of leakage.

The above-mentioned experiments proved that the location and size of leakage can be determined through the flowrate of hall sensors. Through this system, the leakage in subsea pipeline can be detected by an accurate and low-cost method, which is more accurate than the raman distributed fiber sensor, which was mentioned in the introduction section. Additionally, the cost of the system was also lower than the ultrasonic guided wave, optical fiber, and spherical detector, which was also mentioned in the introduction section.

### 3.2. Calculating the Size of Leakage

Meanwhile, the research also used flowrate to calculate the size of the leak. There are two leakage area modes. Type I leakage area is 5.28 cm^2^. Type II leakage area is 1.98 cm^2^. When the actual size of the leak in the pipeline is Type I, the size calculated by the flowrate of the hall sensors is 2.58 cm^2^ on average, as listed in Table 2, indicating that the error between experimental data and theoretical data is 46%. When the actual size of leakage in the pipeline is Type II, the size calculated by the flowrate of the hall sensors is 1.31 cm^2^ on average, as listed in Table 3, indicating that the error between the experimental data and theoretical data is 21%. The cause of this error could be inaccurate flowrate detection resulted from an unstable flow field. The error is acceptable when detecting leakage; however, how to decrease the error in order to make the system more precise is an issue we could address in the future.

## 4. Conclusions

In this study, a real-time monitoring deep-sea pipeline damage sensing system is designed to provide decision-making parameters such as damage coordinates and damage area. The array sensor module is composed of multiple YF-S201 hall sensors and controllers. The flow signal in the deep-sea pipeline can be transmitted to the electronic control interface to analyze the leakage position and leakage flowrate of the pipeline. Through the flowrate of each sensor, the structural safety of the pipeline is judged. After analysis, the location and area of pipeline damage can be determined. The error rate of damage area judgment is as low as 21%. How to decrease the error to make the system more precise is an issue we can address in the future. It is expected to be able to monitor the flow through the array magnetic array sensing module and flow leakage algorithm designed in this study. This can grasp the status of deep-sea pipelines, improve the quality of deep-sea extraction and pipeline maintenance speed, so that the AUV/ROV can accurately locate and perform pipeline maintenance in real time.

## Figures and Tables

**Table 1:** The proportion of flowrate of hall sensor to flowrate of pipeline.

Flowrate of Hall Sensor (L/h)	Flowrate of Pipeline (L/h)	Proportion
32	604	0.053
45	928	0.048
52	1231	0.042
72	1382	0.052
85	1951	0.044

**Table 2:** The result of the size of leakage by the flowrate of sensors for Type I.

Sensor A (L/h)	Sensor B (L/h)	Sensor C (L/h)	Flowrate in the Leakage (cm^3^/s)	Flow Velocity in Leakage (cm/s)	Leakage Area (cm^2^)
82.8	44.5	44.5	226.36	79.03	2.86
67.6	31.5	31.5	213.36	72.66	2.94
43.08	20	20	136.41	69.96	1.95

**Table 3:** The result of the size of leakage by the flowrate of sensors for Type II.

Sensor A (L/h)	Sensor B (L/h)	Sensor C (L/h)	Flowrate in the Leakage (cm^3^/s)	Flow Velocity in Leakage (cm/s)	Leakage Area (cm^2^)
82.8	62	44.5	122.93	79.03	1.56
67.6	50.5	31.5	101.06	72.66	1.39
43.08	31.65	20	67.55	69.96	0.97

## References

[1] Nadzri M.M.M., Ahmad A. Design Issues and Challenges of Long-Range Ultrasonic Testing (LRUT) for Pipeline Inspection. Proceedings of the 12th National Technical Seminar on Unmanned System Technology.

[2] Butterfield J.D., Meruane V., Collins R.P., Meyers G., Beck S.B. (2018). Prediction of leak flow rate in plastic water distribution pipes using vibro-acoustic measurements. Struct. Health Monit..

[3] Atef A., Zayed T., Hawari A., Khader M., Moselhi O. (2016). Multi-tier method using infrared photography and GPR to detect and locate water leaks. Autom. Constr..

[4] Davis P., Brockhurst J. (2015). Subsea pipeline infrastructure monitoring: A framework for technology review and selection. Ocean. Eng..

[5] Ong K., Png W., Lin H., Pua C., Rahman F. Acoustic vibration sensor based on macro-bend coated fiber for pipeline leakage detection. Proceedings of the 2017 17th International Conference on Control, Automation and Systems (ICCAS).

[6] Zhang J., Lian Z., Zhou Z., Song Z., Liu M., Yang K. (2022). Leakage detection in a buried gas pipeline based on distributed optical fiber time-domain acoustic wave signal. Eng. Fail. Anal..

[7] Yi-lai M., Jin-zhong C., Jian L., Guan-nan S. Structure Design of Magnetization Device of Magnetic Leakage Internal Detector for Small-Diameter Oil and Gas Pipeline. Proceedings of the 2021 7th International Symposium on Mechatronics and Industrial Informatics (ISMII).

[8] Frittelli J., Andrews A., Parfomak P.W., Pirog R., Ramseur J.L., Ratner M. (2014). US Rail Transportation of Crude Oil: Background and Issues for Congress.

[9] Li G., Zhu J., Sun R., Lin X., Yang W., Zeng K., Wang F., Wang C., Zhou B. Pipe line safety monitoring using distributed optical fiber vibration sensor in the China west-east gas pipeline project. Proceedings of the Asia Communications and Photonics Conference.

[10] Bolotina I., Borikov V., Ivanova V., Mertins K., Uchaikin S. (2018). Application of phased antenna arrays for pipeline leak detection. J. Pet. Sci. Eng..

[11] Wu Y.-K., Shen S.-C., Lee C.-Y., Chen Y.-J. (2022). A Self-Powered Strain Sensor Applied to Real-Time Monitoring for Movable Structures. Sensors.

[12] Xie Y., Xiao Y., Liu X., Liu G., Jiang W., Qin J. (2020). Time-frequency distribution map-based convolutional neural network (CNN) model for underwater pipeline leakage detection using acoustic signals. Sensors.

[13] Eastvedt D., Naterer G., Duan X. (2022). Detection of faults in subsea pipelines by flow monitoring with regression supervised machine learning. Process Saf. Environ. Prot..

[14] Aloqaily A. (2018). Cross Country Pipeline Risk Assessments and Mitigation Strategies.

[15] Ho M., El-Borgi S., Patil D., Song G. (2020). Inspection and monitoring systems subsea pipelines: A review paper. Struct. Health Monit..

[16] Wang X., Simpson A., Lambert M., Vítkovský J. Leak detection in pipeline systems using hydraulic methods: A review. Proceedings of the Conference on Hydraulics in Civil Engineering, the Institutuion of Engineers.

[17] Hough J.E. (1988). Leak testing of pipelines uses pressure and acoustic velocity. Oil Gas J..

[18] Sood R., Kaur M., Lenka H. (2013). Design and development of automatic water flow meter. Int. J. Comput. Sci. Eng. Appl..

[19] Gosavi G., Gawde G., Gosavi G. Smart water flow monitoring and forecasting system. Proceedings of the 2017 2nd IEEE International Conference on Recent Trends in Electronics, Information & Communication Technology (RTEICT).

[20] Kolhare N., Thorat P. (2013). An approach of flow measurement in solar water heater using turbine flow meter. Int. J. Comput. Technol..

[21] Liu L., Yang L., Gao S. (2022). Propagation Characteristics of Magnetic Tomography Method Detection Signals of Oil and Gas Pipelines Based on Boundary Conditions. Sensors.

[22] Xu T., Zeng Z., Huang X., Li J., Feng H. (2021). Pipeline leak detection based on variational mode decomposition and support vector machine using an interior spherical detector. Process Saf. Environ. Prot..

[23] Kim J., Chae M., Han J., Park S., Lee Y. (2021). The development of leak detection model in subsea gas pipeline using machine learning. J. Nat. Gas Sci. Eng..

[24] Xu Y., Li J., Zhang M., Yu T., Yan B., Zhou X., Yu F., Zhang J., Qiao L., Wang T. (2020). Pipeline leak detection using Raman distributed fiber sensor with dynamic threshold identification method. IEEE Sens. J..

